# Psychometric Properties and Factor Structure of the Polish ChEDE-Q in a Community Sample of Adolescents: Associations with BMI

**DOI:** 10.3390/nu18071028

**Published:** 2026-03-24

**Authors:** Małgorzata Wąsacz, Damian Frej, Danuta Ochojska, Marta Kopańska

**Affiliations:** 1 Department of Medical Communication and Professional Competency Development, Faculty of Medicine, University of Rzeszów, 35-959 Rzeszów, Poland; mwasacz@ur.edu.pl; 2 Department of Automotive Engineering and Transport, Kielce University of Technology, 25-314 Kielce, Poland; dfrej@tu.kielce.pl; 3 Faculty of Health Sciences and Psychology, University of Rzeszów, 35-959 Rzeszów, Poland; dochojska@wp.pl

**Keywords:** ChEDE-Q, child eating questionnaire, eating disorders, children and adolescents, psychometric properties, BMI

## Abstract

Background: The Child and Adolescent Eating Disorder Examination Questionnaire (ChEDE-Q) is a widely used self-report screening instrument for assessing eating disorder psychopathology in young people. Evidence on the psychometric properties of the Polish-language version remains limited. This pilot study evaluated the internal consistency, dimensional structure, and BMI-related convergent validity of the Polish ChE-DE-Q in a regional youth sample. Methods: A cross-sectional design was used, including 200 participants aged 10–18 years. Item characteristics and data quality were examined. Internal consistency was assessed using Cronbach’s alpha and McDonald’s omega. Dimensional structure was evaluated with exploratory factor analysis (EFA) based on a polychoric correlation matrix and confirmatory factor analysis (CFA) comparing one-factor, four-factor, and bifactor models. Convergent validity was examined using Spearman’s rank correlations with BMI and linear regression analyses with BMI z-scores. Results: The global score showed high internal consistency (α = 0.898; ω = 0.900). Subscale reliability ranged from acceptable to high. EFA supported a multidimensional solution. In CFA, the bifactor model showed the best fit among the tested alternatives (CFI = 0.742; TLI = 0.681; RMSEA = 0.122; SRMR = 0.084), but none of the tested models achieved fully satisfactory absolute fit. The global score correlated positively with BMI (rho = 0.282; *p* < 0.001) and was significantly associated with BMI z-score in regression analysis (B = 0.334; *p* < 0.001). Conclusions: The Polish ChEDE-Q global score demonstrated strong internal consistency and preliminary BMI-related convergent validity. The findings provide initial support for a general factor and for using the global score in screening-oriented research; however, the pilot character of the study and the suboptimal absolute fit indices indicate that further validation in larger and more heterogeneous samples is required.

## 1. Introduction

Eating disorders are among the most serious mental disorders due to their somatic and psychosocial consequences, the risk of a chronic course, and, in some cases, elevated mortality [1]. From a public health perspective, what matters is not only the clinical burden, but also the costs borne by patients and families and the loss of quality of life, which at the population level translates into a substantial disease burden [2]. Evidence from systematic reviews indicates that the prevalence of eating disorder-related behaviors and symptoms is high, and in many countries, the problem remains significant even outside clinical populations [3]. In recent years, it has additionally been emphasized that disordered-eating symptoms often emerge already during developmental periods, and their global prevalence among children and adolescents constitutes a growing challenge for prevention and early intervention [4]. At the same time, unfavorable body weight trends persist in child and adolescent populations, including increasing rates of overweight and obesity in many regions worldwide, creating a context of heightened risk for problems related to eating and body image [5].

Contemporary theoretical approaches highlight the transdiagnostic nature of eating disorder psychopathology. In Fairburn’s transdiagnostic model, a key mechanism shared across diagnoses is the overvaluation of weight and shape in self-evaluation, which promotes dietary restraint, binge-eating episodes, and compensatory behaviors, as well as maintaining symptom cycles through cognitive and behavioral mechanisms [6]. From a measurement perspective, this model supports an approach focused on assessing global eating pathology, understood as a common symptom core across diagnostic categories, while simultaneously monitoring clinically salient behaviors such as binge-eating and loss of control.

A particularly important issue in pediatrics and health psychology is the co-occurrence of excess body weight with behaviors typical of eating psychopathology, including binge-eating and eating with a sense of loss of control. A meta-analysis of children and adolescents with overweight and obesity demonstrated a substantial prevalence of such episodes, which is relevant both for prognosis and for intervention planning [7]. At the same time, a systematic review with meta-analysis suggests that obesity treatment with a dietary component may be associated with a risk of developing or exacerbating eating disorder-related behaviors, reinforcing the need for routine monitoring of disordered-eating symptoms in weight reduction programs for minors [8]. Consequently, in both clinical practice and epidemiological research, the use of screening tools with well-documented psychometric properties, appropriate to age and cultural context, becomes crucial.

One of the most commonly used self-report questionnaires for assessing the severity of eating disorder psychopathology is the Eating Disorder Examination Questionnaire, EDE Q. Given the time burden of the full version, shortened forms have been developed, including the EDE QS, designed as a more economical instrument that is also useful for repeated measurement [9]. At the same time, the literature points to considerable variability in findings regarding the factor structure of the EDE Q across samples, which may affect the interpretation of subscales and the global score. Studies in adolescent populations have reported multidimensional solutions, but not always consistently across countries and samples [10]. This is supported by analyses conducted in European and Asian populations, in which the fit of competing models was tested and limited cross-cultural comparability was noted [11,12]. In parallel, newer psychometric approaches, including item response theory analyses, indicate that some items may differ in diagnostic quality and that results may be best interpreted along a continuum with a strong general factor [13]. As a result, in adolescent research, the unidimensionality of the EDE Q has been debated; some data suggest dominance of a general component alongside relatively weaker stability of the classical subscales, which may support prioritizing interpretation of the global score in screening applications and in monitoring change [10,13].

In response to the need to reduce questionnaire length and incorporate content related to bulimic behaviors, other shortened versions have also been developed, such as the EDE Q 13, which combines a brief format with coverage of symptom domains of key clinical importance [14,15]. For screening applications in developmental groups, establishing interpretive thresholds for shortened versions is also important, as demonstrated by studies validating the EDE QS in adolescents and proposing cut-off values for screening [16]. Parallel work has addressed cut-offs in general populations, further enhancing the usefulness of these tools in screening practice [14]. The literature also includes validations of short EDE Q forms in various cultural contexts, incorporating confirmatory factor analysis and Rasch analyses, which additionally underscore the importance of adapting the tool to the language and population [17,18,19]. With respect to clinical interpretation, the need to develop sex-specific discriminative criteria has also been emphasized, as illustrated by work on cut-offs for men [18].

The adaptation and validation of tools for assessing eating disorders spans many language versions and samples with diverse characteristics. For example, evaluation of the psychometric properties of the Malay version of the EDE Q highlights the importance of adaptation procedures and re-verification of reliability and structure in a new context [20]. Studies conducted in Middle Eastern countries have examined correlates of eating psychopathology in local populations, drawing attention to associations with BMI and body dissatisfaction, which are relevant for interpreting convergent validity [21]. A growing line of research also addresses model fit of the EDE Q in groups differing by sexual orientation, including tests of measurement invariance among men [22,23]. Other studies compare alternative brief models in heterogeneous samples, pointing to the field’s rapid development and the risk of interpretive inconsistency in the absence of local validation [24]. In this context, data from men diagnosed with eating disorders are also relevant, as limitations of classical subscale structures can be particularly evident in these populations [25].

Previous studies have shown that EDE-Q interpretation and clinical use may require attention to the characteristics and context of specific underrepresented populations [26,27,28,29,30,31,32]. These findings highlight the importance of cautious generalization across groups; however, as such subgroup-specific issues were not examined in the present study, they are not discussed here in detail.

For child and early adolescent populations, however, what is crucial is not only the fit of norms and cut-offs, but above all the appropriateness of item content and response format. To this end, child-adapted versions of the EDE Q have been developed, including the short form ChEDE Q8, intended to enable an economical assessment of key aspects of eating psychopathology in younger respondents [33]. At the same time, cultural adaptations of the tool are being carried out in different language settings, highlighting the importance of verifying reliability and validity in ethnically and socially diverse samples [34]. Psychometric evaluations of language versions of the ChEDE and ChEDE Q, including comparisons of the interview and questionnaire formats, further demonstrate the need to examine method concordance and subscale properties in clinical populations [35]. In pediatric practice, solutions incorporating caregivers’ perspectives are also promising, as exemplified by validation of the parent version, ChEDE QP, in parent–child dyads [36].

A key issue remains what exactly the ChEDE Q measures, primarily a global eating disorder pathology consistent with the transdiagnostic approach, or four relatively distinct domains analogous to the classical EDE Q subscales: restraint, eating concern, shape concern, and weight concern. On the one hand, observations of high intercorrelations among domains and the primacy of a general factor in some psychometric analyses support interpreting the global score as the most stable indicator of severity [6,13]. On the other hand, in clinical and preventive applications, the domain profile may add value provided that the multidimensional structure is empirically confirmed in a given population and that the subscales show sufficient reliability and discrimination [35,36]. In practice, this implies the need to test competing structural models and to evaluate the utility of the global score and subscales depending on age, sex, BMI, and cultural context.

Against this backdrop, psychometric evaluation of the Polish version of the ChEDE Q among children and adolescents is particularly important. The lack of local data on reliability, item characteristics, factor structure, and convergent validity limits the responsible use of the tool in research and screening practice. For this reason, conducting a pilot psychometric evaluation in a regional population is justified as a step preceding full validation of the instrument in larger and more diverse samples.

## 2. Materials and Methods

This section describes the study design, instrument, variables, and analytical strategy. The research materials and anonymized survey data were deposited in the Zenodo repository. The survey included a demographic section covering age, height, body weight, and place of residence, followed by ChEDE-Q items referring to the previous 28 days.

The pilot analyses focused on the psychometric performance of the 22 scored items and the behavioral indicators covering restrictive eating, food-related preoccupation, loss-of-control eating, compensatory physical activity, and weight- and shape-related concerns

### 2.1. Study Design and Participants

The present study had a cross-sectional, pilot design and aimed to provide a preliminary psychometric evaluation of the Polish version of the Child and Adolescent Eating Disorder Examination Questionnaire (ChEDE-Q) in a regional adolescent sample. Data were collected in 2023–2024 in the Podkarpackie Voivodeship, south-eastern Poland, within the same fieldwork framework and institutional network as the broader regional youth health project. All participants completed the questionnaire once, anonymously, under standardized conditions.

Participants were recruited from three settings representing different socio-health contexts:

- A secondary school in Tyczyn;
- The 2nd Paediatrics, Endocrinology and Paediatric Diabetology Clinic of the Clinical Regional Hospital No. 2 in Rzeszów;
- Zimowit Health Resort in Rymanów-Zdrój.

Recruitment was conducted on site with the cooperation of school staff, clinicians, and personnel involved in youth health promotion activities. Adolescents and their caregivers were informed about the aim and procedure of the study, and those who agreed participated during scheduled assessment sessions organized at the recruitment sites. Because recruitment included both educational and health-related settings, the sample should be interpreted as a regional mixed-setting field sample rather than as a strictly population-representative community sample. This recruitment strategy also helps explain the relatively high BMI values observed in the sample, as adolescents with overweight or obesity were more likely to be represented in the clinical and therapeutic settings.

The target age range for the study was 10–18 years, and only participants within this range were included in the final analyses. This age criterion was applied consistently during data qualification and should be reported uniformly throughout the manuscript.

The inclusion criteria were as follows:

- Age between 10 and 18 years;
- Sufficient reading and comprehension ability to complete the questionnaire independently;
- Provision of parental or legal guardian consent;
- Adolescent’s assent to participate in the study.

The exclusion criteria were:

- Incomplete questionnaires;
- Missing key data required for calculation of questionnaire scores;
- Response patterns indicating insufficient data quality or internal inconsistency;
- After data screening, 200 complete questionnaires were retained for psychometric analyses.

The study was approved by the Bioethics Committee of the District Medical Chamber in Rzeszów (approval no. 78/2022/B) and was conducted in accordance with the Declaration of Helsinki. Participation was voluntary. All participants and their caregivers were informed about the anonymous character of the survey and the possibility of withdrawing at any stage without consequences. Written informed consent was obtained from parents or legal guardians, and assent was obtained from the adolescents prior to participation. Data were collected and stored in accordance with GDPR requirements and were analyzed only in aggregate form.

### 2.2. Study Instrument

The study used the Polish version of the Child and Adolescent Eating Disorder Examination Questionnaire (ChEDE-Q) as a self-report instrument designed to assess eating-related attitudes and behaviors, as well as weight- and shape-related concerns in children and adolescents. The questionnaire refers to experiences from the previous 28 days, which provides a standardized recall period for all symptom-related responses and ensures consistency in the reporting of recent behaviors and attitudes. In the present study, the Polish-language version was administered as the target instrument for a preliminary psychometric evaluation in a regional sample of adolescents.

The questionnaire included both demographic and symptom-related components. The demographic section collected basic participant information, including age, sex, place of residence, and self-reported anthropometric data. Place of residence was classified as rural or urban, while body height and body weight were used for subsequent calculation of BMI-based indicators. The main part of the instrument consisted of items assessing the frequency and severity of core eating disorder-related symptoms over the previous 28 days. Most items were scored using seven response categories reflecting either frequency or intensity, depending on the content of the question. These items covered key domains traditionally associated with eating disorder psychopathology, including dietary restraint, eating concern, shape concern, and weight concern. Additional items referred to dissatisfaction with weight and shape, the influence of weight and shape on self-evaluation, and emotional discomfort in situations involving eating or body exposure. The questionnaire also included supplementary questions concerning selected eating-related episodes and compensatory behaviors, such as episodes of eating accompanied by a sense of loss of control and increased physical activity undertaken in response to concerns about weight gain. In line with the psychometric focus of the present study, the analyses concentrated primarily on the scored ChEDE-Q items contributing to the global score and subscale structure.

Anthropometric measurements were obtained on site by trained research staff. Body height was measured to the nearest 0.1 cm using a SECA 213 stadiometer (Seca GmbH, Hamburg, Germany), and body weight was measured to the nearest 0.1 kg using a SECA 813 electronic scale (Seca GmbH, Hamburg, Germany). Measurements were taken in light clothing and without shoes, twice, and averaged for analysis. BMI was calculated as body weight in kilograms divided by height in meters squared. In addition, BMI z-scores were computed as age- and sex-standardized indicators of relative body mass.

### 2.3. Study Procedure and Variables

The questionnaire was completed once, anonymously, and under standardized conditions at the recruitment sites described above. All participants responded individually and referred to the previous 28 days in accordance with the questionnaire instructions. Before completing the survey, participants received brief procedural guidance to ensure that they understood the general format of the questionnaire, although no assistance was provided in relation to the substantive content of individual answers. The study was designed as a one-wave assessment, and no repeated measurement was performed.

The variables included in the present analyses comprised sociodemographic, anthropometric, and questionnaire-derived psychometric indicators. Sociodemographic variables included age, sex, and place of residence, with residence coded as rural or urban. Anthropometric variables were based on self-reported body height and body weight. These data were used to calculate body mass index (BMI), expressed as body weight in kilograms divided by height in meters squared, and BMI z-scores, treated as age- and sex-standardized indicators of relative body mass. Psychometric variables derived from the ChEDE-Q included item-level scores, subscale scores, and the global score. In the present study, the global score was treated as the principal indicator of overall eating disorder-related psychopathology. Consistent with the aims and scope of this pilot psychometric report, convergent validity was evaluated primarily in relation to BMI and BMI z-score. This analytical decision was made to preserve coherence between the stated objectives of the study, the reported findings, and the limited but clinically relevant set of external variables included in the final analyses.

### 2.4. Data Preparation and Statistical Analyses

Before analyses, data quality control was performed at the item and respondent level, including assessment of response completeness, identification of values outside allowable ranges, and detection of coding inconsistencies. For each item, the proportion of missing data was determined, and response distributions were described using measures appropriate for ordinal data, including the median and interquartile range. Floor and ceiling effects were also evaluated at the item level, defined as the proportion of responses in the extreme categories of the scale, 0 or 6. Additionally, item total correlations were analyzed, corrected for the contribution of a given item to the total score, which enabled a preliminary assessment of each item’s ability to differentiate levels of the measured trait within the sample.

Items rated on the seven-category response scale were recoded to the 0 to 6 range in accordance with the scoring scheme, enabling computation of quantitative scores. The global score and subscale scores were calculated as means of the items comprising a given domain, whereas behavioral items with dichotomous responses were analyzed separately as indicators of behavior frequency and were reported as counts and percentages. Anthropometric variables included body weight, height, and BMI, calculated as body weight in kilograms divided by height in meters squared. In addition, an age- and sex-adjusted BMI z score was computed and treated as an indicator of weight status relative to developmental norms and used in predictive analyses.

Internal consistency was evaluated for the global score and subscales using Cronbach’s alpha and McDonald’s omega, with 95-percent confidence intervals estimated using bootstrap. Factor structure was evaluated in two ways. First, exploratory factor analysis, EFA, was conducted using a polychoric correlation matrix, justified by the ordinal nature of the data, and the number of factors was evaluated based on empirical criteria and the interpretability of the solution. Second, confirmatory factor analysis, CFA, was performed to compare the fit of competing structural models of the instrument, including a one-factor model, a four-factor model, and a bifactor model. Model fit was reported using CFI, TLI, RMSEA, and SRMR, and conclusions were formulated with consideration of the pilot nature of the sample and limitations resulting from item-level response distributions.

Convergent validity was assessed using Spearman rank correlations between questionnaire scores and BMI and other external variables. Between-group comparisons were treated as exploratory and included analyses of differences by sex, age group, and place of residence, with effect sizes reported as the primary approach to interpreting results. Measurement equivalence across sex was additionally evaluated using a multigroup approach, sequentially testing configural, metric, and scalar invariance, and in the case of failure to meet full scalar invariance, a partial scalar approach was considered.

To better highlight the relationship between the severity of symptoms measured by ChEDE Q and weight status, linear regression analysis was conducted, with BMI z score as the dependent variable and the ChEDE Q global score as the predictor. In the basic model, the effect of the global score on BMI z score was estimated, and in a supplementary analysis, age and sex were included as covariates to reduce the risk of confounding due to developmental and sex related differences. Regression coefficients, B, standard errors, 95-percent confidence intervals, *p* values, and model fit, R-squared, were reported. Model assumptions were verified based on residual analysis and assessment of homoscedasticity, and in the case of deviations, robust standard errors were applied.

Exploratory factor analysis was conducted on a polychoric correlation matrix, which is appropriate for ordinal item responses. Confirmatory factor analyses and multigroup confirmatory factor analyses were estimated using the weighted least squares mean and variance-adjusted estimator (WLSMV), recommended for ordered categorical indicators. This approach was selected because the ChEDE-Q items were scored on ordinal response scales and several items showed marked floor and ceiling effects. Confirmatory models were used to compare the one-factor, four-factor, and bifactor solutions. Model fit was evaluated using the Comparative Fit Index (CFI), Tucker–Lewis Index (TLI), Root Mean Square Error of Approximation (RMSEA), and Standardized Root Mean Square Residual (SRMR). In the invariance analyses for ordinal indicators, threshold parameters were examined rather than continuous-item intercepts.

### 2.5. Ethical Aspects and Limitations

The study was survey-based and anonymous, with no collection of data enabling participant identification. Participation was voluntary. Procedures were conducted in accordance with confidentiality principles and ethical standards appropriate for research involving minors, including locally required parental or legal-guardian consent and participant assent appropriate to age and ability to understand the study information. Study limitations follow from its pilot character. The sample was regional, which limits generalization to the nationwide population. No clinical sample was included; therefore, the instrument’s usefulness under diagnostic and therapeutic conditions cannot be directly assessed. Test–retest reliability was not evaluated, which limits inference about measurement stability over time. ROC analysis was not performed; therefore, cut-off points for screening applications were not established. Consequently, the results should be treated as preliminary and requiring confirmation in larger and more diverse samples, ideally including clinical groups and a design allowing assessment of stability and criterion validity.

### 2.6. Practical Application

The Polish version of ChEDE Q can be used as a screening tool in population-based and community studies conducted among children and adolescents, particularly in situations requiring rapid identification of elevated risk of disordered-eating symptoms and an initial assessment of eating psychopathology severity. Given the frequent co-occurrence of excess body weight with loss-of-control eating and other eating disorder-related symptoms, the tool may also be useful in populations with overweight and obesity, for example, in prevention and intervention programs implemented in school settings and within pediatric care. The questionnaire may further support monitoring changes in symptom severity during therapeutic interventions and weight reduction programs, especially when measurement is repeated at subsequent stages, while interpretation should take into account the pilot nature of the available data and the need for further validation, including establishing cut-off points and assessing measurement stability over time.

## 3. Results

### 3.1. Sample Characteristics, Data Quality, and Descriptive Results

A total of 200 complete questionnaires were included in the analysis. Sample characteristics are presented in Table 1.

Descriptive statistics for the ChEDE-Q global score and subscales are presented in Table 2. Overall, body image-related domains showed higher scores than eating-related domains, with the lowest values observed for eating concern.

**Figure 1:**
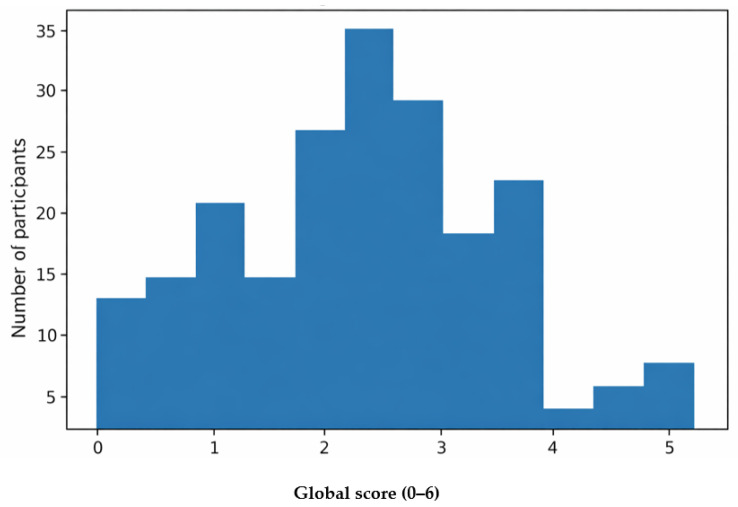
Distribution of the ChEDE Q global score in the study sample.

The distribution of the global score is presented in Figure 1, which indicates that scores were concentrated mainly in the moderate range.

Complete data were available for all 22 scored items; therefore, all psychometric analyses were conducted on the full sample. Item-level response distributions showed floor effects for several items, whereas ceiling effects were less frequent. Corrected item-total correlations varied across items, with the weakest association observed for P2 and the strongest for P24. Detailed item characteristics are presented in Table 3.

### 3.2. Psychometric Properties, Internal Consistency and Factor Structure

Internal consistency for the four subscales and the global score was assessed using Cronbach’s alpha and McDonald’s omega. The global score showed high reliability, whereas subscale reliability ranged from acceptable to high. The strongest reliability was observed for shape concern, while eating concern showed the lowest internal consistency. Detailed results are presented in Table 4.

Exploratory factor analysis was performed using a polychoric correlation matrix. The data were adequate for factor analysis, and parallel analysis supported a three-factor solution. After oblimin rotation, the extracted factors corresponded broadly to body image and self-evaluation, restraint and weight/shape concern, and loss of control/cognitive focus on food. Detailed factor loadings are presented in Table 5.

Correlations among the extracted factors are shown in Table 6. The strongest association was observed between F1 and F2, whereas the correlation between F2 and F3 was negligible.

Convergent validity was evaluated using Spearman’s rank correlations between ChEDE-Q scores and BMI and age. Positive associations with BMI were observed for the global score and most subscales, whereas eating concern was not significantly associated with BMI. Age showed weak negative associations with selected ChEDE-Q domains. Detailed results are presented in Table 7 and Table 8.

A linear regression analysis was performed to examine the relationship between the ChEDE-Q global score and BMI z-score. The global score was a significant predictor in both the univariable and adjusted models. Detailed regression results are presented in Table 9.

### 3.3. Convergent Validity and Additional Analyses

Exploratory analyses examined differences in ChEDE-Q scores by sex, age, and place of residence using the Mann–Whitney U test. No statistically significant sex differences were found for the global score or any of the subscales, and effect sizes were small across domains (Table 10).

For age, the only statistically significant difference was observed for eating concern, which was higher in the older group. No significant differences were found for the remaining domains (Table 11).

No statistically significant differences were found by place of residence, and effect sizes were small across all domains (Table 12).

**Figure 2:**
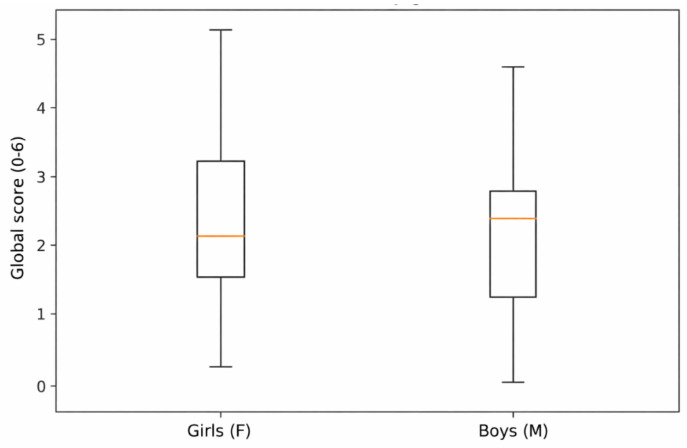
ChEDE Q global score by sex.

To illustrate distributions by sex, a box plot of the global score is presented in Figure 2. Despite visible median differences, distributions largely overlapped, consistent with the nonparametric test results.

**Figure 3:**
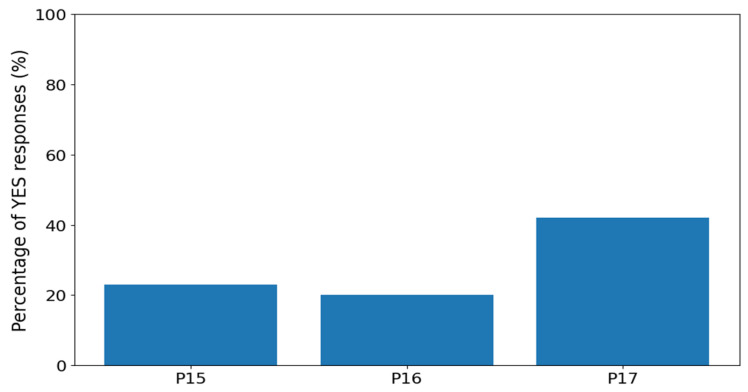
Percentage of YES responses in behavioral items P15 to P17.

Behavioral items P15 to P17 were analyzed as dichotomous variables (yes/no). Response frequencies are presented in Table 13, and their graphical comparison is shown in Figure 3. Increased physical activity undertaken to avoid weight gain was the most frequently endorsed behavior (42.0%), whereas episodes of loss of control over eating (23.0%) and being unable to stop eating (20.0%) were reported less often.

Associations of YES responses in items P15–P17 with the global score and BMI are presented in Table 14. For all three items, the global score was significantly higher in participants endorsing the behavior than in those with NO responses, with the strongest effect observed for P15. For BMI, significantly higher values were found in the YES groups for P15 and P17, whereas no significant difference was observed for P16. Detailed results are presented in Table 14.

**Figure 4:**
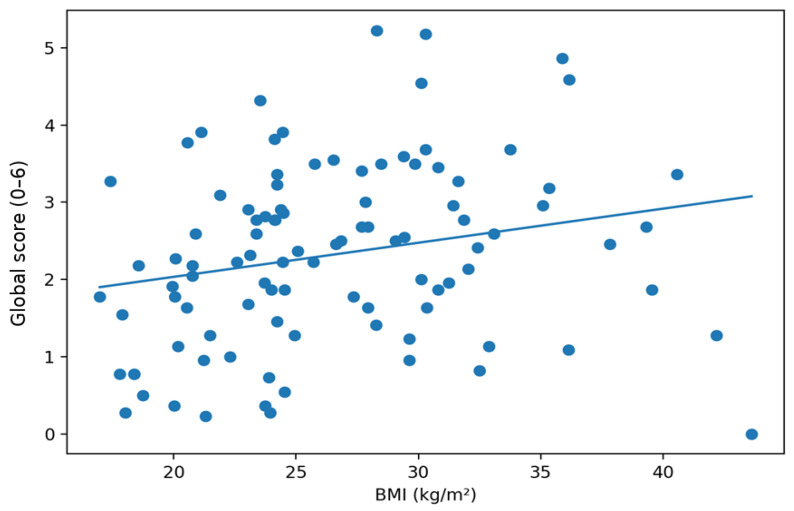
Association between BMI and the ChEDE Q global score.

The relationship between BMI and the global score is illustrated in Figure 4. The plot confirms the positive direction of the association, consistent with the earlier rank correlation results, while also showing substantial dispersion. This means that higher BMI more often co-occurred with higher global severity, but the relationship was not deterministic, and global scores varied even among individuals with similar BMI.

**Figure 5:**
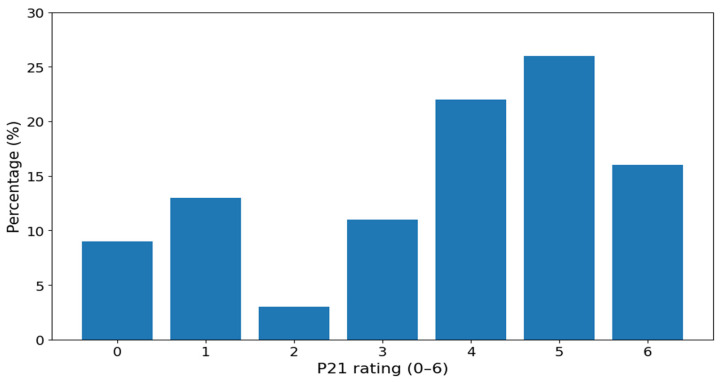
Response distribution for item P21, weight dissatisfaction.

An example of the response distribution for a body image-related item is shown in Figure 5, and selected descriptive characteristics are presented in Table 15.

### 3.4. Factor Structure

To complement the structural analyses, confirmatory factor analysis was performed for the 22 scored items. Three models were tested: a one-factor model, a four-factor model corresponding to the classical domains, and a bifactor model including one general factor and four specific factors. Model fit indices are presented in Table 16.

Overall, the one-factor and four-factor models showed unsatisfactory fit. The bifactor model showed the best relative fit among the tested solutions, although none of the models achieved a fully acceptable fit.

### 3.5. Confirmatory Factor Analysis and Measurement Invariance Across Sex

Measurement invariance by sex was evaluated using multigroup confirmatory factor analysis for the four-factor structure of the instrument. Configural, metric, and scalar invariance were tested hierarchically. Detailed results are presented in Table 17.

The results supported configural and metric invariance across sex. Full scalar invariance was not supported; therefore, a partial scalar invariance model was estimated. The partial scalar model showed fit comparable to the metric model, without further deterioration in fit.

## 4. Discussion

The present pilot study provides preliminary evidence that the Polish version of the ChEDE-Q may be useful as a screening-oriented measure of eating disorder-related psychopathology in children and adolescents aged 10 to 18 years. The most robust result was the strong internal consistency of the global score, which supports its use as the principal overall indicator of symptom severity in research and group-level analyses. In contrast, the subscales were more heterogeneous, with the strongest performance observed for shape concern and weight concern and the weakest for eating concern. This pattern is clinically and psychometrically meaningful, as body- and weight-related concerns may be more salient and more consistently endorsed than narrower eating-related cognitions in mixed and nonclinical developmental samples. Pronounced floor effects observed for several items further support this interpretation, suggesting that less frequent pathological behaviors were uncommon in a substantial part of the sample, whereas body image concerns may represent a more coherent and stable component of the construct.

The reliability profile observed in the present study is broadly consistent with previous research on child- and adolescent-adapted eating disorder questionnaires. The recent Indian adaptation of the ChEDE-Q showed that a shorter two-factor solution centered on eating/restraint concerns and weight/shape concerns performed adequately in adolescents, while the Italian validation study also supported good psychometric performance of the child-adapted instrument in younger populations. Similarly, the ChEDE-Q8 was developed as a brief and psychometrically efficient instrument for children, emphasizing the practical usefulness of a global indicator of eating disorder psychopathology [33,34,35]. Taken together, these findings suggest that, especially in pilot and mixed developmental samples, the global score may be psychometrically more defensible than full reliance on a differentiated subscale interpretation.

The factor-analytic findings should be interpreted with particular caution. Although the bifactor model showed the best relative fit among the tested solutions, none of the tested models achieved fit indices that would typically be considered satisfactory in a full validation study. Accordingly, the present data do not support strong claims about a stable and well-fitting multidimensional latent structure. Rather, they provide preliminary evidence for a meaningful general factor accompanied by specific symptom components that may not be fully separable in a developmental sample. This interpretation is in line with previous adolescent EDE-Q studies, in which the original four-factor structure has often been difficult to replicate. White et al. proposed an alternative adolescent factor solution, while Forsén Mantilla et al. found that the adolescent version of the EDE-Q did not conform well to the standard adult structure and that, in younger respondents, the measure may reflect less differentiated underlying dimensions. More broadly, the systematic review by Jenkins and Rienecke concluded that no consensus currently exists regarding the optimal latent structure of the EDE-Q across samples and settings [11,37,38]. In this context, the present results support using the global score as the most defensible summary index, whereas subscale scores should be interpreted cautiously and primarily as supplementary indicators.

The item-level pattern observed in the present study is also compatible with this interpretation. Several items demonstrated marked floor effects, whereas the strongest item-total relationships were concentrated around body dissatisfaction and the subjective importance of weight and shape. This suggests that body image-related concerns may form the most psychometrically coherent core of the questionnaire in mixed youth samples, whereas more behaviorally specific symptoms may be less frequent and, therefore, less internally cohesive. Similar tendencies toward reduced or reorganized factor structures have been described in adolescent samples from Mexico and Sweden, where parsimonious or modified models performed better than the classical arrangement derived from adult conceptualizations [11,13,39,40]. These findings underline the importance of developmental context when interpreting ChEDE-Q data and suggest that direct transfer of adult structural assumptions to child and adolescent populations may not always be appropriate.

The measurement invariance analyses add value to the study, but they also require a balanced interpretation. Although changes in fit indices across models suggested practical support for metric invariance and partial scalar invariance, the relatively weak fit of the configural model limits the strength of the conclusions that can be drawn. In other words, sex comparisons may be provisionally acceptable, especially at the level of the global score, but the baseline structural uncertainty means that invariance findings should not be overinterpreted. This cautious position is consistent with previous work indicating that some EDE-Q structures may be invariant across sex only partially or under selected model specifications [13,41]. The same caution applies to the observed associations between ChEDE-Q scores and BMI-related indices. The positive association between the global score and both BMI and BMI z-score is theoretically plausible and provides preliminary support for BMI-related convergent validity. However, this effect should not be overstated. In the adjusted model, a substantial portion of explained variance likely reflected age, sex, and sample composition rather than the independent contribution of questionnaire scores alone. Given that the present sample was recruited from mixed educational and health-related settings, and that adolescents with elevated BMI may have been overrepresented, the observed associations should be interpreted as expected cross-sectional covariation rather than as evidence of predictive or diagnostic utility.

Several limitations should be acknowledged. First, the study had a pilot and cross-sectional design, which limits generalizability and precludes conclusions regarding temporal stability or the direction of observed associations. Second, the sample was regional and not population-representative, and the recruitment strategy may have increased the proportion of adolescents with elevated BMI. Third, the anthropometric data were self-reported. Previous work has shown that self-reported height and weight in children and adolescents can correlate strongly with measured values, but may still result in underestimation of weight and BMI and reduced accuracy at the extremes of the distribution [39]. Fourth, although the factor-analytic strategy was ambitious, the CFA results were clearly suboptimal, which reinforces the need for replication in larger and more heterogeneous samples, ideally with explicit use of estimators tailored to ordinal data. Finally, the present study did not include test–retest reliability, criterion validity against interview-based diagnosis, or the development of clinical cut-off values, all of which are essential for full validation and for stronger clinical interpretation.

Despite these limitations, the study makes a meaningful contribution by providing a first multidimensional psychometric evaluation of the Polish ChEDE-Q in a developmental sample. The findings support the use of the global score as the most stable and defensible index at the current stage of validation, while indicating that the subscales require more cautious interpretation. From a practical perspective, the Polish ChEDE-Q may be considered a promising screening-oriented instrument for research and for early risk identification, particularly in groups with elevated BMI, but the present data do not yet justify strong diagnostic conclusions or independent clinical decision-making based solely on questionnaire scores. Future studies should include broader and more-diverse samples, direct anthropometric measurements, formal analyses of temporal stability and criterion validity, and further testing of whether the Polish version is best conceptualized as a predominantly unidimensional measure or as a reduced multidimensional structure more appropriate for children and adolescents [11,13,33,34,35,37,38,39,40,41,42].

The descriptive and behavioral findings complement the psychometric results by indicating that body image-related concerns may represent the most salient component of eating disorder psychopathology captured by the Polish ChEDE-Q in this mixed developmental sample. In contrast, some behaviorally specific items appeared less homogeneous, which is consistent with the more cautious interpretation of subscale scores. Taken together, these findings further support the use of the global score as the most robust summary index at the current stage of validation.

## 5. Conclusions

The Polish version of the ChEDE-Q showed promising psychometric properties in this pilot regional sample. The findings support the global score as the most robust indicator of eating disorder-related psychopathology for screening-oriented research in children and adolescents.

At the same time, the factorial and invariance analyses indicate that the questionnaire structure should be interpreted with caution, particularly at the subscale level. Accordingly, subscale scores should currently be treated as supplementary rather than primary indicators.

Overall, the Polish ChEDE-Q appears to be a promising instrument for early identification of eating disorder-related symptoms, but further validation in larger and more diverse samples is needed before stronger clinical or interpretative conclusions can be drawn.

## Figures and Tables

**Table 1:** Characteristics of the study sample.

Variable	Value
Sample size	200
Gender, girls	108 (54.0%)
Gender, boys	92 (46.0%)
Residence, urban	122 (61.0%)
Residence, rural	78 (39.0%)
Age, years, median [Q1, Q3]	16.5 [14.8, 17.0]
Age, years, mean (SD)	15.68 (2.30)
Height, cm, median [Q1, Q3]	168 [159, 174]
Weight, kg, median [Q1, Q3]	70.0 [60.0, 82.5]
BMI, kg per m^2^, median [Q1, Q3]	24.73 [22.51, 30.30]
BMI, kg per m^2^, mean (SD)	26.59 (5.94)

**Table 2:** Descriptive statistics for ChEDE Q scale scores, 0 to 6.

Score	Median [Q1, Q3]	Mean (SD)	Min to Max
Global score	2.34 [1.52, 3.11]	2.32 (1.17)	0.00 to 5.23
Restraint	1.60 [0.80, 3.00]	1.92 (1.41)	0.00 to 6.00
Eating concern	1.33 [0.67, 2.38]	1.57 (1.13)	0.00 to 4.83
Shape concern	3.00 [1.60, 4.60]	3.02 (1.71)	0.00 to 6.00
Weight concern	2.83 [1.67, 4.00]	2.84 (1.49)	0.00 to 6.00

**Table 3:** Characteristics of scored items, 0 to 6.

Item	Median [Q1, Q3]	Floor n (%)	Ceiling n (%)	Rho, Item Total
P1	2 [0, 3]	54 (27.0%)	26 (13.0%)	0.54
P2	0 [0, 3]	112 (56.0%)	34 (17.0%)	0.18
P3	1 [0, 3]	76 (38.0%)	30 (15.0%)	0.48
P4	2 [1, 4]	36 (18.0%)	36 (18.0%)	0.36
P5	0 [0, 2]	112 (56.0%)	12 (6.0%)	0.51
P6	1 [0, 3]	82 (41.0%)	10 (5.0%)	0.27
P7	0 [0, 2]	104 (52.0%)	14 (7.0%)	0.33
P8	1 [0, 2]	64 (32.0%)	10 (5.0%)	0.29
P9	0 [0, 2]	104 (52.0%)	8 (4.0%)	0.30
P10	2 [0, 6]	60 (30.0%)	52 (26.0%)	0.61
P11	3 [1, 5]	48 (24.0%)	42 (21.0%)	0.64
P12	2 [1, 4]	48 (24.0%)	28 (14.0%)	0.58
P13	3 [1, 6]	40 (20.0%)	64 (32.0%)	0.58
P14	1 [0, 3]	82 (41.0%)	22 (11.0%)	0.65
P18	4 [1, 5]	30 (15.0%)	32 (16.0%)	0.63
P19	4 [2, 5]	32 (16.0%)	26 (13.0%)	0.68
P20	0 [0, 4]	100 (50.0%)	18 (9.0%)	0.37
P21	4 [3, 5]	18 (9.0%)	32 (16.0%)	0.68
P22	4 [1, 5]	30 (15.0%)	30 (15.0%)	0.71
P23	1 [0, 4]	86 (43.0%)	16 (8.0%)	0.45
P24	3 [1, 5]	46 (23.0%)	30 (15.0%)	0.73
P25	4 [1, 5]	42 (21.0%)	30 (15.0%)	0.52

Note: Floor and ceiling indicate the proportion of responses equal to 0 and 6 points, respectively.

**Table 4:** Internal consistency of subscales and the global score, bootstrap 95-percent confidence intervals.

Scale	k	Cronbach’s Alpha	95-Percent CI Alpha	McDonald’s Omega	95-Percent CI Omega
Restraint	5	0.696	[0.618, 0.769]	0.713	[0.642, 0.780]
Eating concern	6	0.651	[0.573, 0.717]	0.657	[0.577, 0.726]
Shape concern	5	0.851	[0.816, 0.890]	0.865	[0.832, 0.898]
Weight concern	6	0.785	[0.734, 0.827]	0.794	[0.747, 0.833]
Global score	22	0.898	[0.877, 0.918]	0.900	[0.879, 0.920]

**Table 5:** Factor loadings after oblimin rotation, values at or above 0.30 shown, empty cells indicate values below 0.30.

Item	F1	F2	F3
P1		0.76	
P2		0.45	
P3		0.57	
P4		0.65	
P5	0.30		0.41
P6			0.39
P7			0.87
P8			
P9			0.36
P10		0.54	
P11		0.71	
P12		0.39	
P13		0.79	
P14	0.31	0.32	0.40
P18	0.80		
P19	0.73		
P20	0.39		
P21	0.68		
P22	0.78		
P23	0.48		0.35
P24	0.87		
P25	0.84		

**Table 6:** Interfactor correlations, oblique rotation.

	F1	F2	F3
F1	1.00	−0.52	−0.25
F2	−0.52	1.00	−0.05
F3	−0.25	−0.05	1.00

**Table 7:** Spearman correlations of ChEDE Q scores with BMI, bootstrap 95-percent confidence intervals.

Score	Spearman Rho	95-Percent CI	*p*
Global score	0.282	[0.143, 0.406]	<0.001
Restraint	0.339	[0.206, 0.459]	<0.001
Eating concern	0.064	[−0.086, 0.205]	0.370
Shape concern	0.214	[0.068, 0.353]	0.002
Weight concern	0.298	[0.155, 0.429]	<0.001

**Table 8:** Spearman correlations of ChEDE Q scores with age, bootstrap 95-percent confidence intervals.

Score	Spearman Rho	95-Percent CI	*p*
Global score	−0.166	[−0.300, −0.031]	0.019
Restraint	−0.082	[−0.217, 0.051]	0.249
Eating concern	0.094	[−0.044, 0.221]	0.187
Shape concern	−0.165	[−0.307, −0.028]	0.019
Weight concern	−0.271	[−0.395, −0.141]	<0.001

**Table 9:** Linear regression, ChEDE Q global score as a predictor of BMI z score; N equals 200.

Model	Predictor	B	SE	95-Percent CI	*p*	R Squared
1	ChEDE Q global score	0.334	0.083	0.172, 0.497	<0.001	0.077
2	ChEDE Q global score, adjusted for age and sex	0.295	0.065	0.167, 0.423	<0.001	0.442
2	Age, years	−0.361	0.032	−0.422, −0.299	<0.001	
2	Male sex, versus female	0.363	0.151	0.068, 0.658	0.016	

**Table 10:** Comparison of ChEDE Q scores by sex, Mann–Whitney U.

Score	n Girls	Median [Q1, Q3] Girls	n Boys	Median [Q1, Q3] Boys	*p*	r_rb	g
Global score	108	2.25 [1.64, 3.36]	92	2.39 [1.27, 2.77]	0.183	0.11	0.24
Restraint	108	1.70 [0.80, 3.00]	92	1.50 [0.80, 2.80]	0.421	0.07	0.19
Eating concern	108	1.50 [0.67, 2.67]	92	1.33 [0.50, 2.00]	0.096	0.14	0.29
Shape concern	108	3.20 [1.80, 4.60]	92	3.00 [1.60, 4.20]	0.222	0.10	0.18
Weight concern	108	3.08 [1.67, 4.17]	92	2.67 [1.67, 3.67]	0.352	0.08	0.14

**Table 11:** Comparison of ChEDE Q scores by age, 13 years or younger versus 14 years or older, Mann–Whitney U.

Score	n 13 or Younger	Median [Q1, Q3] 13 or Younger	n 14 or Older	Median [Q1, Q3] 14 or Older	*p*	r_rb	g
Global score	42	2.77 [1.27, 3.00]	158	2.23 [1.64, 3.26]	0.934	0.01	−0.11
Restraint	42	1.40 [0.60, 2.80]	158	1.60 [0.80, 3.00]	0.296	−0.10	−0.18
Eating concern	42	1.17 [0.33, 1.83]	158	1.50 [0.71, 2.62]	0.016	−0.24	−0.45
Shape concern	42	3.20 [1.20, 4.40]	158	3.00 [1.60, 4.60]	0.872	−0.02	−0.05
Weight concern	42	3.50 [2.00, 4.17]	158	2.67 [1.67, 3.67]	0.138	0.15	0.20

**Table 12:** Comparison of ChEDE Q scores by place of residence, city versus rural, Mann–Whitney U.

Score	n City	Median [Q1, Q3] City	n Rural	Median [Q1, Q3] Rural	*p*	r_rb	g
Global score	122	2.45 [1.64, 3.27]	78	2.23 [1.44, 2.94]	0.254	0.10	0.17
Restraint	122	1.60 [0.80, 3.00]	78	1.40 [0.80, 2.75]	0.663	0.04	0.12
Eating concern	122	1.33 [0.67, 2.17]	78	1.33 [0.71, 2.46]	0.945	−0.01	0.02
Shape concern	122	3.20 [1.60, 4.60]	78	2.80 [1.65, 4.40]	0.155	0.12	0.19
Weight concern	122	2.83 [1.67, 4.17]	78	2.83 [1.75, 3.67]	0.225	0.10	0.21

**Table 13:** Frequency of YES or NO responses in behavioral items.

Item	YES n	YES Percent	NO n	NO Percent
P15	46	23.0	154	77.0
P16	40	20.0	160	80.0
P17	84	42.0	116	58.0

**Table 14:** Associations of behavioral items, YES or NO, with the global score and BMI, Mann–Whitney U.

Item	Variable	n YES	Median [Q1, Q3] YES	n NO	Median [Q1, Q3] NO	*p*	r_rb	g
P15	Global score	46	3.36 [2.56, 3.75]	154	2.00 [1.23, 2.77]	<0.001	0.62	1.27
P15	BMI	46	29.07 [23.63, 31.01]	154	24.44 [22.31, 29.86]	0.013	0.24	0.38
P16	Global score	40	2.93 [2.33, 3.56]	160	2.18 [1.27, 2.88]	<0.001	0.41	0.73
P16	BMI	40	24.72 [23.37, 29.58]	160	24.73 [21.44, 30.31]	0.555	0.06	0.12
P17	Global score	84	2.80 [2.23, 3.45]	116	1.89 [1.27, 2.59]	<0.001	0.43	0.72
P17	BMI	84	28.20 [23.73, 31.63]	116	24.22 [20.76, 28.31]	<0.001	0.31	0.48

**Table 15:** Selected body image and weight concern items, frequencies of extreme responses.

Item	At Least 5 n, Percent	0 n, Percent	Median [Q1, Q3]
P21	84 (42.0)	18 (9.0)	4 [3, 5]
P22	72 (36.0)	30 (15.0)	4 [1, 5]
P24	54 (27.0)	46 (23.0)	3 [1, 5]
P25	52 (26.0)	42 (21.0)	4 [1, 5]
P13	74 (37.0)	40 (20.0)	3 [1, 6]
P11	64 (32.0)	48 (24.0)	3 [1, 5]

**Table 16:** CFA model fit for 22 items, N = 200.

Model	χ^2^	df	CFI	TLI	RMSEA	SRMR
One-factor	1048.69	209	0.607	0.565	0.142	0.105
Four-factor	916.15	203	0.666	0.620	0.133	0.100
Bifactor	737.78	187	0.742	0.681	0.122	0.084

**Table 17:** Sex invariance tests in MG CFA for the four-factor model, N = 200.

Model	Constraints	χ^2^ (df)	CFI	TLI	RMSEA	SRMR	ΔCFI, ΔRMSEA, ΔSRMR
1	Configural	1694.944 (402)	0.537	0.468	0.127	0.123	not applicable
2	Metric	1739.040 (424)	0.530	0.487	0.125	0.132	−0.008, −0.002, +0.009
3	Scalar	1795.359 (446)	0.517	0.500	0.123	0.133	−0.012, −0.002, +0.000
4	Partial scalar	1754.602 (442)	0.530	0.509	0.122	0.132	+0.001, −0.003, +0.000

## Data Availability

All data and materials are included in the manuscript.

## Nomenclature

P1	Have you tried to eat less in order to change your body shape or weight?
P2	When going to bed, did you refrain from getting up at night and eating anything for 8 h?
P3	Have you tried not to eat foods you like in order to change your body shape or weight?
P4	Have you tried to follow dietary rules to change your weight or body shape, for example amount, calories, what and when you eat?
P5	Have you wanted your stomach to be completely empty?
P6	Difficulty concentrating because of thoughts about food?
P7	Fear that you might lose control over your eating behaviors?
P8	Overeating, how often did you eat too much?
P9	Have you eaten in secret?
P10	Have you wanted your stomach to be completely flat?
P11	How often have you thought about not gaining weight?
P12	How often have you felt heavy?
P13	How often have you thought about losing weight?
P14	How often have you felt guilty after eating?
P15	During overeating, did you feel a lack of control over what and how much you ate, no or yes plus frequency.
P16	Have you had moments when you felt you could not stop eating, no or yes plus frequency.
P17	Have you exercised a lot in order not to gain weight, no or yes plus frequency.
P18	Does your weight influence the way you think about yourself?
P19	Does the shape of your body influence the way you think about yourself?
P20	Would weighing yourself once a week for the next month be a problem for you?
P21	Have you been dissatisfied with your weight?
P22	Have you been dissatisfied with your body shape?
P23	Have you worried that others would see you eating?
P24	Have you felt uncomfortable or shy when seeing your own body, for example, in a mirror or while bathing?
P25	Have you felt uncomfortable or shy when others could see your body, for example, in changing rooms or while swimming?

## References

[1] Treasure J., Duarte T.A., Schmidt U. (2020). Eating disorders. Lancet.

[2] van Hoeken D., Hoek H.W. (2020). Review of the burden of eating disorders: Mortality, disability, costs, quality of life, and family burden. Curr. Opin. Psychiatry.

[3] Galmiche M., Déchelotte P., Lambert G., Tavolacci M.P. (2019). Prevalence of eating disorders over the 2000–2018 period: A systematic literature review. Am. J. Clin. Nutr..

[4] López-Gil J.F., García-Hermoso A., Smith L., Firth J., Trott M., Mesas A.E., Jiménez-López E., Gutiérrez-Espinoza H., Tárraga-López P.J., Victoria-Montesinos D. (2023). Global proportion of disordered eating among children and adolescents: A systematic review and meta-analysis. JAMA Pediatr..

[5] NCD Risk Factor Collaboration (NCD-RisC) (2017). Worldwide trends in body-mass index, underweight, overweight, and obesity from 1975 to 2016: A pooled analysis of 2416 population-based measurement studies in 128.9 million children, adolescents, and adults. Lancet.

[6] Fairburn C.G., Cooper Z., Shafran R. (2003). Cognitive behaviour therapy for eating disorders: A “transdiagnostic” theory and treatment. Behav. Res. Ther..

[7] He J., Cai Z., Fan X. (2017). Prevalence of binge and loss of control eating among children and adolescents with overweight and obesity: A meta-analysis. Int. J. Eat. Disord..

[8] Jebeile H., Gow M.L., Baur L.A., Garnett S.P., Paxton S.J., Lister N.B. (2019). Treatment of obesity, with a dietary component, and eating disorder risk in children and adolescents: A systematic review with meta-analysis. Obes. Rev..

[9] Gideon N., Hawkes N., Mond J., Saunders R., Tchanturia K., Serpell L. (2016). Development and psychometric validation of the EDE-QS, a short form of the Eating Disorder Examination Questionnaire (EDE-Q). PLoS ONE.

[10] Carey M., Kupeli N., Knight R., Troop N.A., Jenkinson P.M., Preston C. (2019). Eating Disorder Examination Questionnaire (EDE-Q): Norms and psychometric properties in U.K. females and males. Psychol. Assess..

[11] Forsén Mantilla E., Birgegård A., Clinton D. (2017). Factor analysis of the adolescent version of the Eating Disorders Examination Questionnaire (EDE-Q): Results from Swedish general population and clinical samples. J. Eat. Disord..

[12] Mitsui T., Yoshida T., Komaki G. (2017). Psychometric properties of the Eating Disorder Examination Questionnaire (EDE-Q) in Japanese adolescents. Biopsychosoc. Med..

[13] Dufour R., Steiger H., Booij L. (2025). Examining dimensionality and item-quality of the Eating Disorder Examination Questionnaire in individuals with eating disorders using item response theory analysis. Int. J. Eat. Disord..

[14] Meule A., Hilbert A., de Zwaan M., Brähler E., Koch S., Voderholzer U. (2024). Cutoff scores of the Eating Disorder Examination-Questionnaire for the German population. Int. J. Eat. Disord..

[15] Lev-Ari L., Bachner-Melman R., Zohar A.H. (2021). Eating Disorder Examination Questionnaire (EDE-Q-13): Expanding on the short form. J. Eat. Disord..

[16] Dahlgren C.L., Lichtenstein M.B., Brockmeyer T., Hansen S., Clausen L. (2025). Identifying a cut-off score for the Eating Disorder Examination Questionnaire Short Form (EDE-QS) in adolescents. BMC Psychol..

[17] He J., Sun S., Fan X. (2021). Validation of the 12-item Short Form of the Eating Disorder Examination Questionnaire in the Chinese context: Confirmatory factor analysis and Rasch analysis. Eat. Weight Disord..

[18] Schaefer L.M., Smith K.E., Leonard R., Wetterneck C., Smith B., Farrell N., Riemann B.C., Frederick D.A., Schaumberg K., Klump K.L. (2018). Identifying a male clinical cutoff on the Eating Disorder Examination-Questionnaire (EDE-Q). Int. J. Eat. Disord..

[19] Lichtenstein M.B., Clausen L. (2021). Validation of the Eating Disorder Examination Questionnaire in Danish: A confirmatory factor analytic and Rasch-based approach. J. Clin. Med..

[20] Taib M.N., Abdul Khaiyom J.H., Fauziana R. (2021). Psychometric properties of the Malay version of the Eating Disorder Examination Questionnaire (EDE-Q). Eat. Behav..

[21] Melisse B., van Furth E.F., de Beurs E. (2022). Eating disorder examination questionnaire (EDE-Q): Validity and norms for Saudi nationals. Eat. Weight Disord..

[22] Scharmer C., Donahue J.M., Heiss S., Anderson D.A. (2020). Factor structure of the Eating Disorder Examination-Questionnaire among heterosexual and sexual minority males. Eat. Behav..

[23] Klimek P., Murray S.B., Brown T., Gonzales M., Blashill A.J. (2021). Measurement invariance of the Eating Disorder Examination-Questionnaire across sexual orientation in men. Int. J. Eat. Disord..

[24] Compte E.J., Brown T.A., Lavender J.M., Murray S.B., Nagata J.M. (2023). Evaluation of alternative brief models of the Eating Disorder Examination-Questionnaire across diverse samples. J. Eat. Disord..

[25] Laskowski N.M., Eriksson J., Monell E., Birgegård A., Forsén Mantilla E. (2023). Psychometric evaluation of the Eating Disorder Examination-Questionnaire in men with eating disorders. J. Eat. Disord..

[26] Nagata J.M., Murray S.B., Compte E.J., Pak E.H., Schauer R., Flentje A., Capriotti M.R., Lubensky M.E., Lunn M.R., Obedin-Maliver J. (2020). Community norms for the Eating Disorder Examination Questionnaire (EDE-Q) among transgender men and women. Eat. Behav..

[27] Nagata J.M., Compte E.J., Cattle C.J., Flentje A., Capriotti M.R., Lubensky M.E., Murray S.B., Obedin-Maliver J., Lunn M.R. (2020). Community norms for the Eating Disorder Examination Questionnaire (EDE-Q) among gender-expansive populations. J. Eat. Disord..

[28] Nagata J.M., Compte E.J., Murray S.B., Schauer R., Pak E., Flentje A., Capriotti M.R., Lubensky M.E., Lunn M.R., Obedin-Maliver J. (2021). Community norms for the Eating Disorder Examination Questionnaire (EDE-Q) among cisgender bisexual plus women and men. Eat. Weight Disord..

[29] Avila J.T., Golden N.H., Aye T. (2019). Eating disorder screening in transgender youth. J. Adolesc. Health.

[30] Roberts S.R., Maheux A.J., Watson R.J., Puhl R.M., Choukas-Bradley S. (2022). Sexual and gender minority (SGM) adolescents’ disordered eating: Exploring general and SGM-specific factors. Int. J. Eat. Disord..

[31] Austen E., Greenaway K.H., Griffiths S. (2020). Differences in weight stigma between gay, bisexual, and heterosexual men. Body Image.

[32] Thorne N., Witcomb G.L., Nieder T., Nixon E., Yip A., Arcelus J. (2019). A comparison of mental health symptomatology and levels of social support in young treatment seeking transgender individuals who identify as binary and non-binary. Int. J. Transgenderism.

[33] Kliem S., Schmidt R., Vogel M., Hiemisch A., Kiess W., Hilbert A. (2017). An 8-item short form of the Eating Disorder Examination-Questionnaire adapted for children (ChEDE-Q8). Int. J. Eat. Disord..

[34] Ahuja L., Diedrichs P.C., Garbett K.M., Chaudhry A., Hasan F., Uglik-Marucha N., Vitoratou S., Dhillon M., Shroff H., Lewis-Smith H. (2023). Adaptation and validation of the Child Eating Disorder Examination Questionnaire (ChEDE-Q) for Use in English among Adolescents in Urban India. Nutrients.

[35] Bonvini L., Taddei S., Caini S., Calugi S., Bugli G., Tarchi L., Chiari S., Galli I., Giunti I., Marino C. (2025). Child eating disorder examination (ChEDE) interview and child eating disorder examination questionnaire (ChEDE-Q): Psychometric properties of the Italian versions. Eat. Weight Disord..

[36] Lange C., Schmidt R., Hilbert A. (2025). Validation of the parent version of the eating disorder examination-questionnaire adapted for children in parent-child dyads with children with and without loss of control eating. J. Eat. Disord..

[37] White H.J., Haycraft E., Goodwin H., Meyer C. (2014). Eating disorder examination questionnaire: Factor structure for adolescent girls and boys. Int. J. Eat. Disord..

[38] Jenkins P.E., Rienecke R.D. (2022). Structural validity of the Eating Disorder Examination-Questionnaire: A systematic review. Int. J. Eat. Disord..

[39] Rand-Giovannetti D., Cicero D.C., Mond J.M., Latner J.D. (2020). Psychometric Properties of the Eating Disorder Examination-Questionnaire (EDE-Q): A Confirmatory Factor Analysis and Assessment of Measurement Invariance by Sex. Assessment.

[40] Rios-Leyvraz M., Ortega N., Chiolero A. (2022). Reliability of Self-Reported Height and Weight in Children: A School-Based Cross-Sectional Study and a Review. Nutrients.

[41] Penelo E., Negrete A., Portell M., Raich R.M. (2013). Psychometric Properties of the Eating Disorder Examination Questionnaire (EDE-Q) and Norms for Rural and Urban Adolescent Males and Females in Mexico. PLoS ONE.

[42] Decaluwé V., Braet C., Fairburn C., Beglin S. (1999). Child Eating Disorder Examination—Questionnaire.

